# Circulating and Tumor-Infiltrating Immune Checkpoint-Expressing CD8^+^ Treg/T Cell Subsets and Their Associations with Disease-Free Survival in Colorectal Cancer Patients

**DOI:** 10.3390/cancers14133194

**Published:** 2022-06-29

**Authors:** Alhasan Alsalman, Mohammad A. Al-Mterin, Khaled Murshed, Ferial Alloush, Samia T. Al-Shouli, Salman M. Toor, Eyad Elkord

**Affiliations:** 1 Natural and Medical Sciences Research Center, University of Nizwa, Nizwa 616, Oman; hasan.alsalman95@gmail.com (A.A.); mohammed.mterin@unizwa.edu.om (M.A.A.-M.); 2 Department of Pathology, Hamad Medical Corporation, Doha P.O. Box 3050, Qatar; kmurshed@hamad.qa (K.M.); falloush@hamad.qa (F.A.); 3 Pathology Department, Immunology Unit, College of Medicine, King Saud University, Riyadh P.O. Box 2925, Saudi Arabia; salshouli@ksu.edu.sa; 4 College of Health and Life Sciences (CHLS), Hamad Bin Khalifa University (HBKU), Qatar Foundation (QF), Doha P.O. Box 34110, Qatar; mstoor@hbku.edu.qa; 5 Biomedical Research Center, School of Science, Engineering and Environment, University of Salford, Manchester M5 4WT, UK

**Keywords:** CD8^+^ T cells, TILs, PBMCs, Progression-Free Survival, immune checkpoints, Tregs

## Abstract

**Simple Summary:**

Colorectal cancer is one of the leading causes of global cancer-related mortality. Tumor-infiltrating effector immune cells play critical roles in tumor control, and their activity can dictate disease outcomes. In this study, we provide evidence of the associations between different CD8^+^ T cell subpopulations with disease-free survival (DFS) in CRC patients. We report associations between higher levels of certain circulating and tumor-infiltrating CD8^+^ T cell subsets and improved clinical outcomes in CRC patients.

**Abstract:**

T cells in the tumor microenvironment (TME) have diverse roles in anti-tumor immunity, including orchestration of immune responses and anti-tumor cytotoxic attack. However, different T cell subsets may have opposing roles in tumor progression, especially in inflammation-related cancers such as colorectal cancer (CRC). In this study, we phenotypically characterized CD3^+^CD4^-^ (CD8^+^) T cells in colorectal tumor tissues (TT), normal colon tissues (NT) and in circulation of CRC patients. We investigated the expression levels of key immune checkpoints (ICs) and Treg-related markers in CD8^+^ T cells. Importantly, we investigated associations between different tumor-infiltrating CD8^+^ T cell subpopulations and disease-free survival (DFS) in CRC patients. We found that FoxP3 expression and ICs including PD-1, CTLA-4, TIM-3, and LAG-3 were significantly increased in tumor-infiltrating CD8^+^ T cells compared with NT and peripheral blood. In the TME, we found that TIM-3 expression was significantly increased in patients with early stages and absent lymphovascular invasion (LVI) compared to patients with advanced stages and LVI. Importantly, we report that high levels of certain circulating CD8^+^ T cell subsets (TIM-3-expressing, FoxP3^−^Helios^−^TIM-3^+^ and FoxP3^−^Helios^+^TIM-3^+^ cells) in CRC patients were associated with better DFS. Moreover, in the TME, we report that elevated levels of CD25^+^ and TIM-3^+^ T cells, and FoxP3^+^Helios^−^TIM-3^+^ Tregs were associated with better DFS.

## 1. Introduction 

Colorectal cancer (CRC) is among the main causes of global cancer-related mortality, responsible for around 8% of overall deaths attributed to cancer [1,2]. Tumor immune surveillance is dependent on various immune effector cells, which are involved in the recognition and killing of tumor cells [3]. Elevated infiltration of lymphocytes in rectal or colon tumor tissues is mostly associated with prolonged patient survival, while immunosuppressive factors favor tumor development and progression [3].

Although patient survival is affected by multiple factors including disease stage and histological grading, several studies have highlighted the significance of the immune system on patient survival [4]. The dynamic interactions involving tumor-infiltrating lymphocytes (TILs) and tumor cells enabled the development of immune classification of solid tumors termed ‘Immunoscore’, which assesses in situ T cell infiltrates for utilization as a prognostic and predictive tool [5]. Importantly, Immunoscore classification has been validated as a robust tool for predicting recurrence risk in CRC patients [6]. CD8^+^ cytotoxic T cells have antitumor activities; a higher CD8^+^ T cell density has been associated with better clinical outcomes [7]. In contrast, T regulatory cells (Tregs) are one of the most difficult obstacles in anti-tumor immunity and successful anti-cancer immunotherapy [8]. Notably, recent studies have reported that, in addition to CD4^+^ Tregs, CD8^+^ Tregs could have prominent roles in tumor immune evasion. Additionally, tumor-infiltrating CD8^+^ Tregs show synergistic immunosuppression with CD4^+^ Tregs [9]. CD8^+^CD25^+^FoxP3^+^ cells are significantly elevated in blood and tissue of CRC patients, and exhibit suppressive activity against CD4^+^CD25^−^ T-cell proliferation and Th1-cytokine production [3,4]. Additionally, CD8^+^Helios^+^ T cells are elevated in peripheral blood mononuclear cell (PBMCs) and tumor-infiltrating lymphocytes (TILs) of CRC liver metastases, but the precise role of Helios remains largely unknown in anti-tumor immunity [10].

Immune checkpoints (ICs) maintain immune homeostasis via fine-tuning the extent of immune activation and the prevention of autoimmunity [11]. Different types of cancers are able to induce over-expression of ICs in various immune cell populations, leading to increases in IC inhibitory signals and immune evasion [12]. For instance, the presence of CD8^+^PD-1^+^ T cells in the tumor microenvironment (TME) was correlated with reduction in cytokine and perforin production [13]. Additionally, the overexpression of TIM-3 restricts T-cells’ responses, and levels of circulating and tumor-infiltrating CD8^+^PD-1^+^TIM-3^+^ cells, which produced significantly less IFN-γ than CD8^+^PD-1^−^TIM-3^−^ cells, were increased in CRC patients [14]. Overall, while various studies have reported relationships between elevated IC expression in the tumor microenvironment (TME) with evidence of disease prognosis in various cancers [15], continued efforts are warranted to decipher the impact of imbalances in various IC-expressing T cell subsets on disease outcomes.

The role of less-conventional CD8^+^ Tregs in cancer progression has been recently highlighted [16]. There are no reliable markers to distinguish CD8^+^ Tregs from conventional CD8^+^ T cells. Notably, FoxP3 is expressed in both CD8^+^ T cells and CD4^+^CD25^+^ T cells in humans, although its expression is significantly higher in CD4^+^ T cells. Thus far, FoxP3 can be considered as the most reliable marker for identification of CD4^+^ and CD8^+^ Tregs [17]. In this study, we investigated CD3^+^CD4^−^ T cells, which predominantly comprise CD8^+^ T cells, in colorectal tumor tissues (TT), and compared them with normal colon tissues (NT) and peripheral blood. We aimed to investigate the expression levels of important ICs and markers associated with Tregs. Importantly, we also explored the associations between different CD8^+^ T cell subpopulations and disease-free survival (DFS) in CRC patients to highlight their impact on clinical outcomes. Findings of this study present TIM-3 as a biomarker for improved DFS in CRC patients.

## 2. Materials and Methods

### 2.1. Sample Collection

This study was executed under ethics approval from Hamad Medical Corporation, Doha, Qatar (study ref. MRC-02-18-012). All subjects included in this study were cancer-treatment naïve and submitted written informed consent before donating samples. CD8^+^ T cells were identified as CD3^+^CD4^−^ T cells, as we have previously described in several studies [10,18,19,20]. Details of patients included in the flow cytometric analyses are described in Toor et al. [18]. Briefly, peripheral blood was collected from 34 CRC patients, while tissue samples (NT and TT) were collected from 27 out of these 34 patients because some NT and TT failed to yield enough cells for flow cytometric analyses. PBMCs were separated from fresh blood using density gradient centrifugation. PBMCs and tissue samples were frozen in freezing media for subsequent analyses, as previously described [18].

DFS data for the clinical outcomes were retrieved after completion of sample collection. Interpretable data were obtained for 32 PBMCs and 22 NILs/TILs. The follow-up survival period for all patients included was calculated {median 160.5 weeks (95% CI 151.1–171.0)}. Four patients exhibited progressive disease, as tumor recurrence (locally) or by the presence of newly developed lymph node and/or distant metastasis. A contrast-enhanced computed tomography (CT) of the chest, abdomen, and pelvis was conducted during clinical follow-up to assess disease progression. The clinical and pathologic characteristics of study subjects included in DFS analysis are described in Table 1.

### 2.2. Multi-Parametric Flow Cytometry Analyses

PBMCs and cells separated from NT and TT were stained for flow cytometric analyses, as previously described [18]. All data were generated on a BD LSRFortessa X-20 flow cytometer (BD Biosciences) and analysis performed on FlowJo software (v.10; FlowJo, Ashland, OR, USA).

### 2.3. Statistical Analyses

The statistical analyses were conducted on GraphPad Prism software (v.9; GraphPad Software, San Diego, CA, USA). Statistical significance in grouped analyses was determined by Kruskal–Wallis test. Paired/unpaired *t*-tests or Wilcoxon matched-pairs signed rank test/Mann–Whitney tests were utilized based on distribution of data as assessed by the Shapiro–Wilk normality test, for comparisons within and between groups.

The different immune cell subsets were divided into low and high groups based on variances from the mean (normally distributed data) or median (non-normally distributed data). The Kaplan–Meier approach was utilized for prediction of DFS, while the log-rank test was used for evaluation of differences in DFS among groups.

## 3. Results 

### 3.1. CD8^+^ T Cell Subsets in Colorectal Cancer and Association with DFS

**Figure 1:**
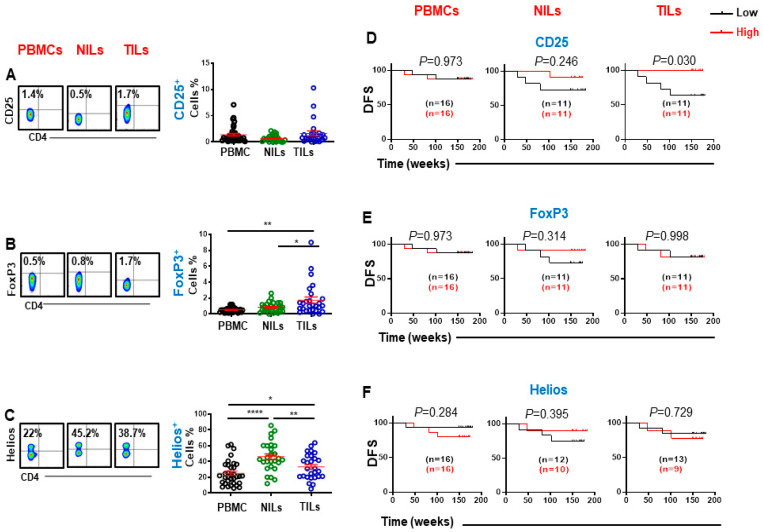
Flow cytometric plots, scatter plots and Kaplan–Meier survival curves for DFS based on the levels of CD25-, FoxP3-, and Helios-expressing CD3^+^CD4^−^ T cells. Representative flow cytometric plots and scatter plots present CD25 (**A**), FoxP3 (**B**) and Helios (**C**) expression in CD8^+^ T cells in PBMCs, NILs and TILs. CRC patients with high levels of CD25 (**D**), FoxP3 (**E**) and Helios (**F**) expressing CD8^+^ T cells were compared with patients with low levels of these cells to determine DFS. The number of patients included in each comparison are specified on each survival curve. Statistical analysis are shown with significance levels indicated at * *p* < 0.05, ** *p* < 0.01, and **** *p* < 0.0001.

We investigated levels of CD25, FoxP3, and Helios expression in CD3^+^CD4^−^ (CD8^+^) T cells in periphery and in tissues of CRC patients. We found that CD25 was expressed at low levels on CD8^+^ T cells, compared to CD4^+^ T cells as previously reported [18]. Moreover, there were no significant differences in CD25 expression on CD8^+^ T cells in the TME and peripheral blood (Figure 1A). Importantly, we found that CD8^+^FoxP3^+^ Tregs were higher in the colorectal TME and were rarely detected in circulation or in normal tissues. (PBMC; 0.5 ± 0.1 vs. NILs; 0.8 ± 0.1 vs. TILs; 1.7 ± 0.4, Figure 1B). Interestingly, there was significantly higher Helios expression in CD8^+^ T cells in normal colon tissue, compared with tumor tissue and circulation (25.2 ± 2.6 vs. 46.1 ± 3.6 vs. 33.2 ± 3.0, Figure 1C). Higher Helios expression on NILs compared to TILs prompted us to investigate and compare FoxP3 and Helios co-expression on CD8^+^ T cells. We found that Tregs in normal colon tissues comprise of significantly higher proportions of stable Tregs (FoxP3^+^Helios^+^) compared to tumor tissues (Supplementary Figure S1). These results suggest that CD8^+^ Tregs within the TME may represent induced Treg phenotypes, which may be less stable due to low Helios expression. However, further investigations are warranted to determine their stability within the TME.

We then investigated the associations between these cell subsets and DFS. We found that high levels of tumor-infiltrating CD8^+^CD25^+^ T cells were significantly associated with longer DFS, while there was no association between the level of these cells and DFS in PBMCs and NILs (Figure 1D). In contrast, FoxP3 and Helios expression on CD8^+^ in PBMCs, TILs, and NILs were not associated with DFS in CRC patients (Figure 1E,F).

### 3.2. Immune Checkpoints and Association with Progression-Free Survival

**Figure 2:**
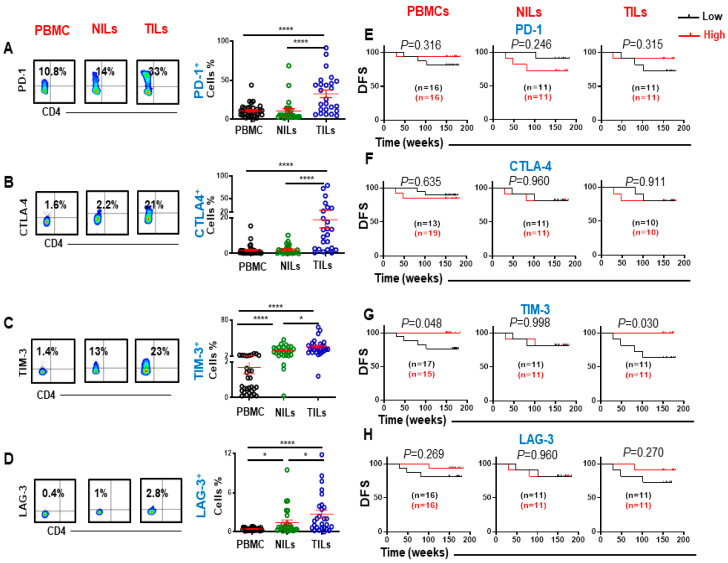
Flow cytometric plots, scatter plots, and Kaplan–Meier survival curves for DFS based on the levels of PD-1, CTLA-4, TIM-3, and LAG-3-expressing CD3^+^CD4^−^ T cells. Representative flow cytometric and scatter plots present the levels of PD-1(**A**), CTLA-4 (**B**), TIM-3 (**C**), and LAG-3 (**D**) expressing CD8^+^ T cells in PBMCs, NILs, and TILs. CRC patients with high levels of PD-1 (**E**), CTLA-4 (**F**)*,* TIM-3 (**G**), and LAG-3 (**H**)-expressing cells were compared with patients with low levels of these cells to determine DFS. The number of patients included in each comparison are specified on each survival curve. Statistical analysis are shown with significance levels indicated at * *p* < 0.05 and **** *p* < 0.0001.

**Figure 3:**
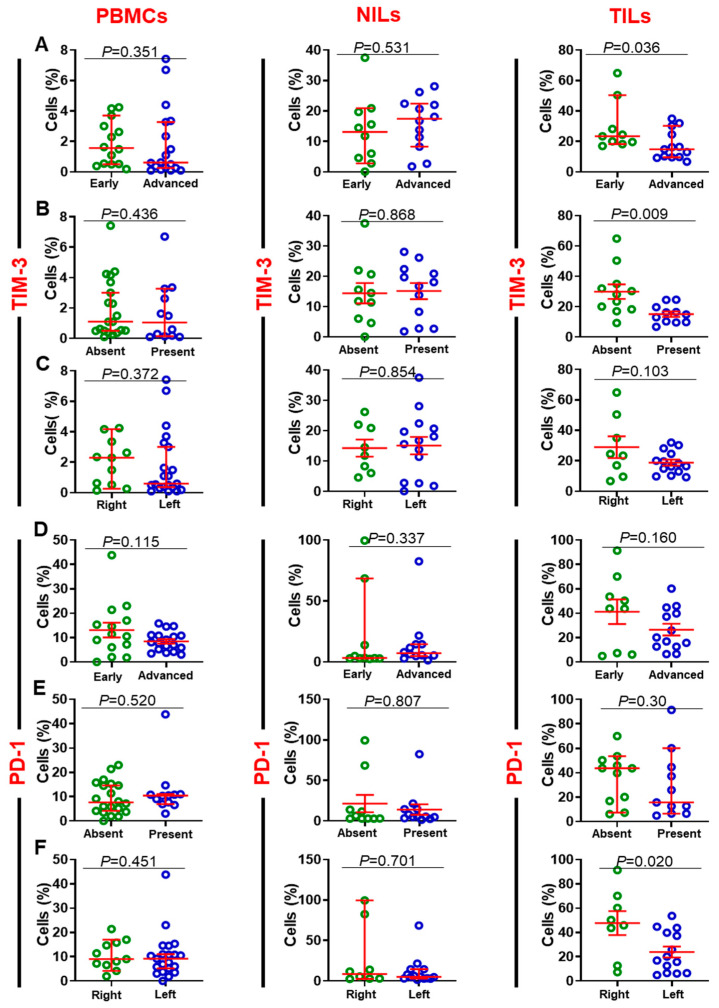
Comparison of TIM-3 and PD-1 expression on CD3^+^CD4^−^ T cells in PBMCs, TILs and NILs. CRC patients were grouped based on disease stages (early; stage I/II and advanced; stage III/IV), anatomical location (right-sided and left-sided) and absent/present LVI. Scatter plots present the differences in the levels of TIM-3-expressing cells in early and advanced stages (**A**), absent/present LVI (**B**), right-sided and left-sided tumors (**C**), and levels of PD-1 in early and advanced stages (**D**), absent/present LVI (**E**), and right-sided and left-sided tumors (**F**) in PBMCs, NILs, and TILs.

Next, we investigated ICs expression on CD8^+^ T cells in circulation, NT and TT. Similar to ICs expression on CD4^+^ T cells [18], PD-1, CTLA-4, TIM-3, and LAG-3 were also overexpressed on CD8^+^ TILs (Figure 2A,D). Moreover, CD8^+^PD-1^+^, CD8^+^CTLA-4^+^, and CD8^+^TIM-3^+^ T cells showed higher expression in the TME compared to CD8^+^LAG-3^+^ T cells (PD-1: PBMC; 10.9 ± 1.4 vs. NILs; 10.5 ± 3.0 vs. TILs 32.6 ± 4.9, CTLA-4: 1.8 ± 0.5 vs. 2.1 ± 0.5 vs. 18.8 ± 4.5, TIM-3: 1.7 ± 0.3 vs. 13.2 ± 1.8 vs. 22.2 ± 2.9, & LAG-3: 0.4 ± 0.1 vs. 1.4 ± 0.4 vs. 2.7 ± 0.6, Figure 2A,D). We then determined the associations between IC-expressing CD8^+^ T cells and DFS. There was no association between the levels of CD8^+^PD-1^+^, CD8^+^CTLA-4^+^, and CD8^+^LAG-3^+^ with DFS in both PBMCs and TILs in CRC patients (Figure 2E,F,H). However, patients with higher levels of CD8^+^TIM-3^+^ in circulation and TILs, but not in NILs, showed significantly longer DFS (Figure 2G). We then grouped patients in our cohort based on pathologic stages (early and advanced). Early-stage colon cancer (stage I/II) represents patients with completely resected tumors and no subsequent evidences of the involvement of adjacent organs, lymph nodes, or distant sites [21]. In contrast, late-stage CRC (stage III/IV) is defined as locally advanced inoperable or metastatic CRC [22]. We then performed a sub-analysis for these groups by comparing the expression of different immune markers. There were no significant variances in the expression levels of FoxP3, CD25, CTLA-4, and LAG-3 in CD8^+^ T cells among patients with early and advanced stages. Interestingly, we found that the expression level of TIM-3 in TILs, but not in PBMCs and NILs, was significantly higher in patients with early stages than advanced stages {median (95% CI); 23.4 (16.9–42.4) vs. 14.9 (11.7–23.4), *p* = 0.036} (Figure 3A). Additionally, PD-1 expressions in circulation and the TME, but not in NILs, were higher in patients with early stages than advanced stages {mean ± SEM; PBMC: 13.1 ± 3.0 vs. 8.4 ± 0.9, *p* = 0.115; TILs: 41 ± 10.1 vs. 26.5 ± 4.7, *p* = 0.160} (Figure 3D). We then focused our investigation on comparing the expression of TIM-3 and PD-1 markers in CD8^+^ T cells based on absence/presence of lymphovascular invasion (LVI), and anatomical location (left-sided and right-sided). Right-sided CRC emerge in cecum, ascending colon, hepatic flexure and/or transverse colon, whereas left-sided CRC emerge in the splenic flexure, descending, and/or sigmoid colon [23]. We found that TIM-3 expression in TILs, but not in PBMCs and NILs, was significantly higher in patients without LVI than those with LVI {mean ± SEM; TILs: 29.9 ± 4.8 vs. 25.1 ± 1.8, *p* = 0.009} (Figure B). Moreover, we did not observe any differences in the expression levels of PD-1 between patients with LVI absent/present (Figure 3E). When patients were divided into two groups based on anatomical location, we found that expression levels of TIM-3 in the right-sided CRC patients in TILs, but not in PBMCs and NILs, was higher than left-sided CRC patients {mean ± SEM; TILs: 29.0 ± 7.1 vs. 18.8± 2.0, *p* = 0.103} (Figure 3C). Additionally, expression levels of PD-1 in TILs, but not in PBMCs and NILs, in the right-sided were significantly higher than left-sided CRC patients {mean ± SEM; 47.7 ± 9.8 vs. 23.9 ± 4.5, *p* = 0.02} (Figure 3F).

### 3.3. Expression of Immune Checkpoints on FoxP3^−^Helios^+/−^ CD8^+^ T cells 

**Figure 4:**
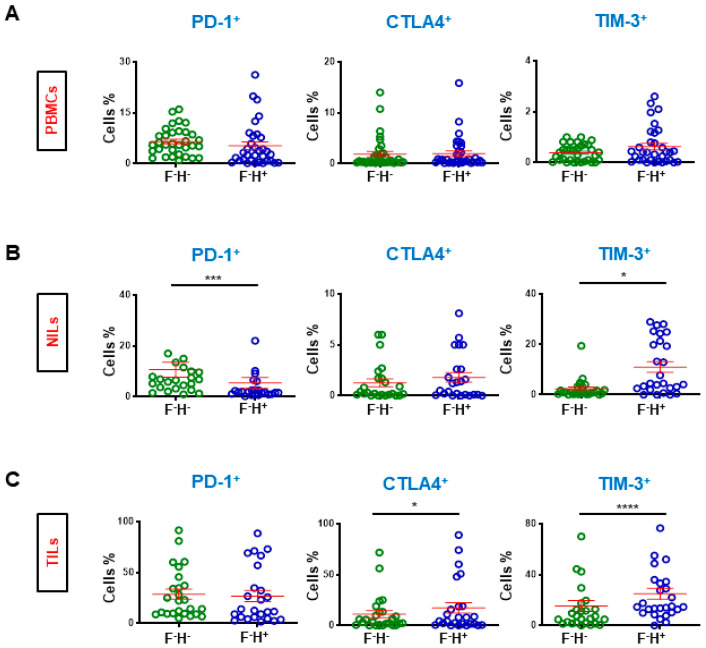
Comparison of different immune checkpoint expression on FoxP3^−^Helios^−^ and FoxP3^−^Helios^+^ in CD3^+^CD4^−^ T cells. Patients were divided based on the expression of Helios. Scatter plots show the differences in levels of immune checkpoint expression on CD8^+^FoxP3^−^Helios^−^ and CD8^+^FoxP3^−^Helios^+^ T cells in PBMCs (**A**), NILs (**B**), and TILs (**C**). Statistical analysis are shown with significance levels indicated at * *p* < 0.05, *** *p* < 0.001 and **** *p* < 0.0001.

Due to low FoxP3 expression on CD8^+^ T cells, we investigated if ICs (PD-1, CTLA-4, and TIM-3) are mainly expressed on FoxP3^−^Helios^+^ or FoxP3^−^Helios^−^ CD8^+^ T cells in periphery, normal tissue and in the TME (Figure 4). Additionally, due to low LAG-3 expression on CD8^+^ T cells, we did not study its expression on these cell subsets in the TME, periphery, and normal tissue. We did not find any differences for ICs expression in Helios^+^ and Helios^−^ CD8^+^ T cells in circulation (Figure 4A). However, PD-1 showed significantly higher expression on CD8^+^FoxP3^−^Helios^−^ NILs than CD8^+^FoxP3^−^Helios^+^ NILs (10.7 ± 2.9 vs. 5.4 ± 2.1, Figure 4B), while no significant differences were recorded in CTLA-4 on FoxP3^−^Helios^−^ and FoxP3^−^Helios^+^ expressing on CD8^+^ NILs (1.3 ± 0.4 vs. 1.8 ± 0.5), but TIM-3 was predominantly expressed on CD8^+^FoxP3^−^Helios^+^ NILs (2.3 ± 0.8 vs. 11.0 ± 2.1) (Figure 4B). Importantly, no significant differences were recorded in PD-1 expression on CD8^+^FoxP3^-^Helios^-^ or on CD8^+^FoxP3^-^Helios^+^ TILs, but TIM-3 and CTLA-4 showed significantly higher expression on CD8^+^Helios^+^ TILs (CTLA-4: 11.1 ± 3.5 vs. 17.2 ± 5.1 & TIM-3: 15.4 ± 4.2 vs. 25.0 ± 4.2, Figure 4C).

### 3.4. Association of IC-Expressing CD8^+^ T Cells with DFS 

**Figure 5:**
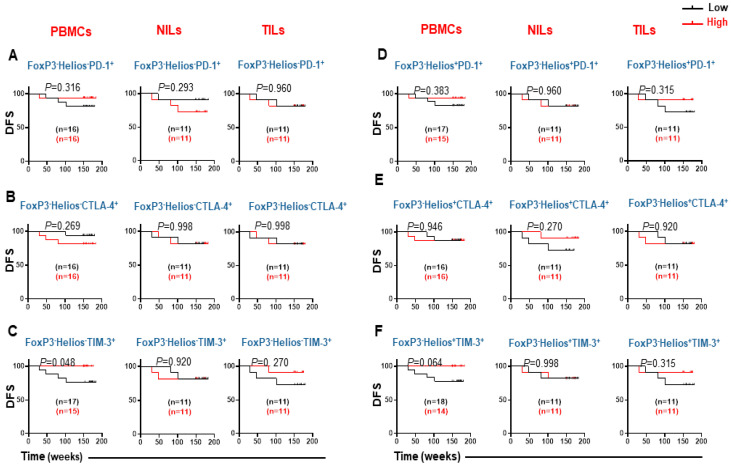
Kaplan–Meier survival curves for DFS based on the levels of FoxP3^−^Helios^−^ and FoxP3^−^Helios^+^ CD3^+^CD4^−^ T cells expressing PD-1, CTLA-4, and TIM-3. CRC patients with high levels of FoxP3^−^Helios^−^ PD-1^+^ (**A**), CTLA-4^+^ (**B**), TIM-3^+^ (**C**), and FoxP3^−^Helios^+^ PD-1^+^ (**D**), CTLA-4^+^ (**E**), and TIM-3^+^ (**F**) cells were compared with CRC patients with low levels of these cells to determine DFS. The number of patients included in each comparison are specified on each survival curve.

We determined which immune checkpoints, expressed on CD8^+^ T cell subpopulations, based on FoxP3 and Helios expression, can be associated with DFS. There were no associations between the levels of circulating and tumor-infiltrating CD8^+^FoxP3^−^Helios^−^PD-1^+^ with DFS (Figure 5A). Additionally, high levels of circulating CD8^+^FoxP3^−^Helios^−^ CTLA-4-expressing T cells indicated a trend with poor DFS, whereas no such potential associations were recorded between these cells and DFS in NT and TT (Figure 5B). Interestingly, increased levels of circulating CD8^+^FoxP3^−^Helios^−^ TIM-3-expressing T cells were significantly associated with prolonged DFS. Furthermore, high levels of CD8^+^FoxP3^−^Helios^−^TIM-3^+^ in TILs showed a trend towards better DFS (Figure 5C).

CD8^+^FoxP3^−^Helios^+^PD-1^+^ and CD8^+^FoxP3^−^Helios^+^CTLA-4^+^ did not show any associations with DFS in PBMCs, NILs, or TILs (Figure 5D,E). Notably, patients with elevated levels of circulating CD8^+^FoxP3^−^Helios^+^TIM-3^+^ T cells tended to survive longer than patients with lower levels of these cells (Figure 5F). However, there was no association between levels of CD8^+^FoxP3^−^Helios^+^TIM-3^+^ and DFS in TILs and NILs (Figure 5F).

**Figure 6:**
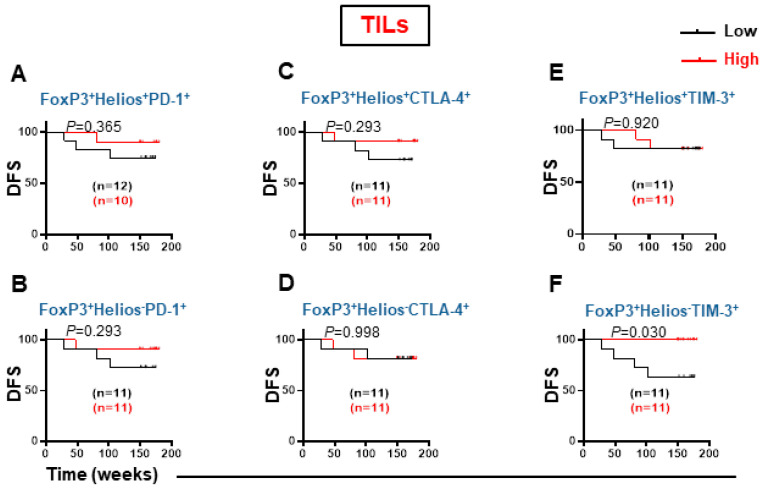
Kaplan–Meier survival curves for DFS based on the levels of FoxP3^+^Helios^+^ and CD3^+^CD4^−^FoxP3^+^Helios^−^ CD3^+^CD4^−^ T cells expressing PD-1, CTLA-4, and TIM-3 in TILs. FoxP3^+^Helios^+^PD-1^+^ (**A**), FoxP3^+^Helios^−^PD-1^+^ (**B**), FoxP3^+^Helios^+^CTLA-4^+^ (**C**), FoxP3^+^Helios^−^CTLA-4^+^ (**D**), FoxP3^+^Helios^+^TIM-3^+^ (**E**), and FoxP3^+^Helios^−^TIM-3^+^ (**F**) cell subsets were grouped into low and high groups, and DFS determined. The number of patients included in each comparison are specified on each survival curve.

Higher FoxP3 expression on CD8^+^ TILs prompted us to investigate associations between FoxP3 and Helios-expressing CD8^+^ TILs with DFS (Figure 6). There were no association between levels of CD8^+^FoxP3^+^Helios^+^PD-1^+^, CD8^+^FoxP3^+^Helios^−^PD-1^+^, CD8^+^ FoxP3^+^Helios^+^CTLA-4^+^, and CD8^+^FoxP3^+^Helios^−^CTLA-4^+^ with DFS in TILs (Figure 6A,D). Interestingly, patients with high level of CD8^+^FoxP3^+^Helios^−^TIM-3^+^ in tumor tissue had significantly improved DFS (Figure 6F). Otherwise, there no association was recorded between the levels of CD8^+^FoxP3^+^Helios^+^TIM-3^+^ with DFS in TILs (Figure 6E).

**Figure 7:**
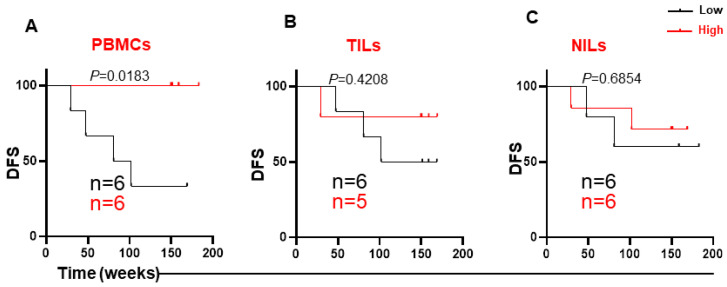
Kaplan–Meier curves of DFS based on levels of CD3^+^CD4^−^TIM-3^+^ in PBMCs, TILs, and NILs in the group of patients with LVI. Patients in the presence of LVI were divided into high and low groups for TIM-3 expression in PBMCs (**A**), TILs (**B**), and NILs (**C**), and DFS was determined for these groups. The number of patients included in each comparison are specified on each survival curve.

Given that TIM-3 expression showed a significant association with DFS, we then performed sub-analyses for different TIM-3-expressing immune cell subsets, depending on the anatomical location, TNM stages and LVI absent/present. The most significant data were obtained from the LVI sub-analysis. Four out of the twelve patients with LVI had disease progression. Interestingly, none of the patients with high TIM-3 expression in circulation had disease progression, and higher levels of TIM-3 in PBMCs, but not in TILs or NILs, were associated with longer DFS (Figure 7A,C). The median survival of CRC patients with low TIM-3 expression in PBMCs was 91.4 weeks.

## 4. Discussion

Tumor-infiltrating lymphocytes in solid tumors predominantly comprise CD3^+^ T cells. Changes in the levels, location, or functional status of T cells may thus have an impact on tumor outcomes [24]. Some studies reported that cytotoxic CD8^+^ T cells have antitumor activities; therefore, elevated levels of tumor-infiltrating CD8^+^ T cells were correlated with longer survival [25,26]. The alpha chain (CD25) of the IL-2 receptor is the most widely known cellular-activation marker [27], while CD8^+^CD25^+^ T cells have characteristics of central memory-like cells [28]. We found accumulation of CD25^+^CD8^+^TILs in CRC. However, the emerging roles of CD8^+^ Tregs in tumor immune evasion [29], led us to investigate these cells in CRC TME. CD8^+^CD28^−^ Tregs reside in majority of human tumors and can inhibit proliferation and cytotoxicity of T cells [30]. Importantly, CD8^+^CD25^+^FoxP3^+^ Tregs were present at significantly higher levels in the TME and circulation of CRC patients [4]. We report that, compared to NT, FoxP3 was expressed at significantly higher levels, while Helios was expressed at lower levels in CD8^+^ T cells in the TME. FoxP3-expressing CD8^+^ Tregs have previously been reported to effectively suppress naïve T cell proliferation in prostate cancer [31]. CD8^+^FoxP3^+^ T cells could be classified as a tightly regulated population that shares developmental and behavioral characteristics with conventional CD4^+^FoxP3^+^ Tregs but lacks potent suppressive action [32]. However, another study reported that CD8^+^FoxP3^+^ Tregs demonstrated strong immunosuppressive properties in CRC [4]. The CD8^+^FoxP3^+^ Treg subtype constitutes a minor proportion of the overall Tregs [26]. Of note, FoxP3 is upregulated on activated T cells, and data from functional studies will ascertain the suppressive capacities of these cells. Moreover, although previous studies have reported CD8^+^ Tregs with this phenotype are present in majority of CRC tumors [33], their overall levels were significantly lower than CD4^+^ Tregs [18]. In addition, Helios is a crucial transcription factor, which stabilizes Tregs in the face of inflammatory reactions [34], and Helios-dependent STAT5 activation is also required for CD8^+^ Treg survival and in inhibiting terminal T cell differentiation [34]. We found reduction in levels of Helios-expressing CD8^+^ TILs. Overall, our results demonstrate that the low levels of tumor-infiltrating CD8^+^ Teff cells are dominated by CD4^+^ and CD8^+^ Tregs in CRC tumors, which display activated phenotypes with immunosuppressive functions [18].

CD8^+^FoxP3^+^ Tregs have a suppressive role in different types of cancers such as prostate, colorectal, hepatic and gastric cancers, similar to CD4^+^FoxP3^+^ T cells [4,35,36,37]. Transient FoxP3 expression during CD8^+^ T cell activation could prevent excessive immunological activation and resulting damage at inflammation sites [37,38]. Yoon et al., reported that the high level of FoxP3^+^ Tregs was associated with a positive effect on survival only in colon cancer patients with low levels of CD8^+^ T cell infiltration [39]. However, the survival rates were best in patients with a high density of CD8^+^ or FoxP3^+^ T cells, and lowest survival rates were observed with a low expression of both markers. These findings imply that CD8^+^ and FoxP3^+^ T cells may work together to control the anti-tumor immunity [39]. Our results showed that expression of FoxP3 in CD8^+^ in PBMCs, TILs and NILs were not associated with DFS in CRC patients. These findings should be confirmed in a larger number of patients.

CD3^+^CD4^−^ T cells also comprise additional T cell subsets at low levels within the TME, most notably γδ T cells which are typically associated with favorable outcomes due to their roles in anti-tumor immunity alongside CD8^+^ TILs [40]. The fundamental advantage γδ T cells have over αβT cells in the context of tumor immunity is the MHC-unrestricted recognition of tumor antigens [41]. In contrast, IL-17-producing γδ T cell subsets exhibit immunosuppressive and pro-tumor roles [42]. Notably, IL-17-producing γδ T cells also promote the influx of other immunosuppressive cells such as myeloid-derived suppressor cells (MDSCs) and their levels correlate with advanced stage CRC [43]. In relation to negative regulation of anti-tumor immunity via IC expression, γδ T cells rarely express CTLA-4 but PD-1 can be expressed on activated cells, while TIM-3 expression has been shown to reduce the cytotoxicity of Vγ9Vδ2 T cells [42,44]. We found high IC expression on CD3^+^CD4^−^ TILs, which shows the high proportion of CD8^+^ TILs in our identified populations but the presence of low levels of γδ T cell subsets cannot be fully mitigated.

Tumor-infiltrating CD8^+^ T cells highly express different ICs in different types of cancer [15]. PD-1 pathway inhibits effector immune responses [2]. It is expressed on various immune cells including T cells, B cells, NKT cells monocytes, and macrophages [45]. Moreover, Level of CD8^+^PD-1^+^ T cells was significantly increased in the TME of CRC patients, compared to CD8^+^ T cells in tumor-free lymph nodes [13]. In agreement with this, we found that PD-1 was expressed at significantly higher levels on CD8^+^ T cells in the TME, compared with PBMCs and NILs. Some studies reported that high levels of CD8^+^PD1^+^ T cells were associated with poor clinical outcomes in patients with different cancers such as breast, renal cell carcinoma, and nasopharyngeal carcinoma [46,47,48]. Moreover, elevated PD-1 expression in TT was linked with poor prognosis of CRC patients with stage I-III cancer [49]. On the other hand, another study reported that a hightened PD-1 expression was associated with superior prognosis in CRC patients [50]. Inomata et al., found that CD4^+^PD-1^+^ T cell levels, but not CD8^+^PD1^+^ or CD8^+^CTLA-4^+^, predict longer PFS in non-small cell lung cancer patients undergoing different IC inhibitor treatments [51]. In our study, we found that expressions of PD-1 on CD8^+^ in TILs, PBMCs, and NILs were not associated with DFS in CRC patients. However, these findings should be confirmed in larger number of patients.

TIM-3 has been identified as one of the critical factors in regulating T cells responses [19]. Many studies reported that TIM-3 expression in different types of cancer such as gastric cancer, cervical, and colon cancers was significantly increased in tumor tissues [52,53,54]. In line with these studies, we also reported that TIM-3 expression was significantly higher in TILs, compared with NILs and PBMCs. Moreover, TIM-3 is expressed, not only on different immunocytes, but also in a variety of cancer cells [55]. TIM-3 function in CRC remains largely unclear [54]. Elevated TIM-3 expression was linked with poor prognosis in solid tumors [54]. Zhou et al., reported that high TIM-3 expression was linked with shorter survival in CRC patients [54]. Moreover, Sun et al.,. reported that low expression of TIM-3 could increase invasion and metastasis in CRC [56]. However, in renal cell carcinoma, Zhang et al., reported that TIM-3 expression was associated with improved PFS and overall survival in primary or metastatic tumors [57]. We report that high levels of circulating TIM-3^+^, FoxP3^−^Helios^+^TIM-3^+^ and FoxP3^−^Helios^−^TIM-3^+^ CD8^+^ T cell subsets were associated with prolonged DFS, suggesting their potential anti-tumor roles in CRC. Moreover, high levels of tumor-infiltrating TIM-3^+^ and FoxP3^+^Helios^−^TIM-3^+^ CD8^+^ Tregs were associated with better DFS. Some studies promote the inhibitory roles of TIM-3 in suppressing effector Th1/Tc1 responses [14,58]; therefore, TIM-3 could have beneficial anti-inflammatory role in the CRC TME. Of note, Li et al., showed that TIM-3 is also highly expressed on γδ T cells, which represent a low population of CD3^+^CD4^−^CD8^−^ T cell populations in the TME, in CRC patients, and attributed reduction in perforin/granzyme B production in γδ T cell subtypes to TIM-3 expression [44]. Therefore, associations between TIM-3 expression and improved DFS in CD8^+^ and FoxP3-expressing CD8^+^ TILs provide evidence for its immunomodulatory roles, which may be beneficial in limiting tumor progression.

LAG-3 protein is expressed in tumor-infiltrating lymphocytes but not in other CRC cells [59]. In different types of cancer, such as lung, breast, stomach, ovarian, and colon cancers, LAG3-expressing T cells were increased in TILs [59,60,61,62]. LAG-3 expression in TILs was significantly associated with improved DFS in patients with stage II colon cancer [63]. Moreover, increased expression of LAG-3 on CD8^+^ TILs was associated with better PFS in liver metastases colorectal cancer patients [64]. Our study showed that levels of CD8^+^LAG-3^+^ cells were significantly increased in the TME, compared with peripheral blood and normal tissue. Moreover, we found that expression levels of LAG-3 on CD8^+^ T cells in TILs, PBMCs, and NILs were not associated with DFS in CRC patients.

Studies have reported that CRC patients with MSI-H tumors exhibit improved prognoses compared to patients with MSS tumors [65,66]. After controlling for pathological characteristics, it has been found that the survival rate of CRC patients with MSI is 15% higher compared to MSS CRC [67]. Moreover, stage II colorectal cancer with MSI-H has been found to have a better prognosis. However, in advanced stages, this issue remains controversial [68]. Notably, MSI-H tumors contain a higher proportion of CD8^+^ T cells and a higher percentage of M1 macrophages compared to MSI-L CRCs [69]. In addition PD-1 and CTLA-4 levels were also significantly raised in dMMR/MSI-H compared to dMMR/MSI-L and MSS CRC tumors [70]. In our study, we were not able to conduct such comparisons, as only four patients in our study cohort had dMMR/MSI-H (Table 1). Patients with dMMR/MSI-H tumors constitute a low proportion among CRC patients [71]. Of note, removing these four MSI-H patients did not affect the results for DFS, but this limitation of our study may be considered in future investigations performed in larger cohorts comprising higher numbers of MSI-H cases.

Based on gene-expression analyses for molecular stratification, CRC tumors are also divided into consensus molecular subgroups (CMS) [72]. There are substantial differences in the biological foundation of each CMS subtype; 1 (MSI immune), 2 (canonical), 3 (metabolic) and 4 (mesenchymal) [72,73]. This molecular classification is one of the more robust classifications for CRC, and can decipher the link between the genetic makeup and the tumor immune landscape, with potential prognostic significance. However, till present, it is not widely adopted and does not have an impact in clinical decision-making, especially for metastatic CRC [73]. The classification is based on gene expression and transcriptome subtyping, which we could not perform at this stage of our study, which therefore may constitute one of the limitations of this study.

By analyzing data from The Cancer Genome Atlas (TCGA), Kitsou et al., investigated associations between the genetic and immune profiles of CRC tumors and explored their effects on clinical outcomes [69]. Authors reported that *LAG3* was significantly downregulated, whereas *IDO1* was overexpressed in CRC. Notably, higher expressions of *CTLA4* and *PD1* were associated with improved survival, while higher TIL load correlated with *CTLA4*, *HAVCR2* (TIM-3), *LAG3,* and *CD274* (PD-L1) expressions in colon adenocarcinoma. Authors concluded that CRC patients with higher IC expression and immunogenic mutations are more likely to benefit from the respective immune checkpoint inhibition [69]. Our findings provide additional evidence in identifying one of the specific IC-expressing T cell populations, which are associated with improved DFS in CRC patients.

## 5. Conclusions

It is noteworthy that many studies reported associations for several ICs in bulk tumor tissues with prognoses, but did not determine such associations on the T cell level. To our knowledge, this study is the first to report significant associations between high levels of circulating and tumor-infiltrating CD8^+^TIM-3^+^ T cell subsets and longer DFS in CRC patients. Our data demonstrate that TIM-3 expression in CD8^+^ T cells is a potential biomarker for improved DFS in CRC patients. Overall, identification of the exact T cell subpopulations contributing to clinical outcomes is critical for prognoses and therapeutic targeting.

## Figures and Tables

**Table 1:** Characteristic features of colorectal cancer patients.

	CRC Patients
**Number**	32 {22} §
**Median age {range}**	61 {31–96}
**Gender** {Male: Female}	23:9
**TNM stage**	
I	5 {1} §
II	9 {8} §
III	15 {11} §
IV	3 {2} §
**Tumor budding**	
Low	11{7} §
Intermediate	11{7} §
High	10 {8} §
**Right/Left-sided**	
Left	21 {14} §
Right	11 {8} §
**Tumor histological type**	
Adenocarcinoma NOS	30 {20} §
Mucinous	2 {2} §
**Lymphovascular invasion** **(LVI)**	
Yes	12 {11} §
No	20 {11}§
**Molecular testing**	
KRAS mutations	4 {2} §
BRAF mutations	1 {1} §
**MSI-H/dMMR**	4 {3} §

CRC: Colorectal cancer; MSI-H/dMMR: High MicroSatellite Instability/deficient MisMatch Repair. § Samples used for analyses of tumor-infiltrating lymphocytes.

## Data Availability

All data generated or analyzed in this study are available upon reasonable request.

## Supplementary Materials

The following supporting information can be downloaded at: https://www.mdpi.com/article/10.3390/cancers14133194/s1, Figure S1: FoxP3 and Helios co-expression on CD8^+^ T cells in CRC patients.

## Abbreviations

CRC	Colorectal cancer
CTLA-4	Cytotoxic T-lymphocyte-associated protein 4
FoxP3	Forkhead box P3
ICs	Immune checkpoints
IDO1	Indoleamine 2,3-Dioxygenase 1
LAG-3	Lymphocyte-activation gene 3
NILs	Normal tissue-infiltrating lymphocytes
NT	Normal tissues
PBMC	Peripheral blood mononuclear cell
PD-1	Programmed cell death-1
DFS	Disease-free survival
TCGA	The Cancer Genome Atlas
TILs	Tumor-infiltrating lymphocytes
TIM-3	T-cell immunoglobulin and mucin domain-3
TME	Tumor microenvironment
Treg	T regulatory cell
TT	Tumor tissues
