# Supplementary material for: Circulating and Tumor-Infiltrating Immune Checkpoint-Expressing CD8+ Treg/T Cell Subsets and Their Associations with Disease-Free Survival in Colorectal Cancer Patients
Source: Cancers (Basel). 2022 Jun 29;14(13):3194. doi: 10.3390/cancers14133194 (PMC9265020; doi:10.3390/cancers14133194)
Supplement: Supplementary file 1 [file cancers-14-03194-s001.zip › cancers-1775717-supplementary.pdf]

Supplementary Materials

Circulating and Tumor-Infiltrating Immune Checkpoint-Expressing CD8⁺ Treg/T Cell Subsets and Their Associations with Disease-Free Survival in Colorectal Cancer Patients

Alhasan Alsalman, Mohammad A. Al-Mterin, Khaled Murshed, Ferial Alloush, Samia T. Al-Shouli, Salman M. Toor and Eyad Elkord

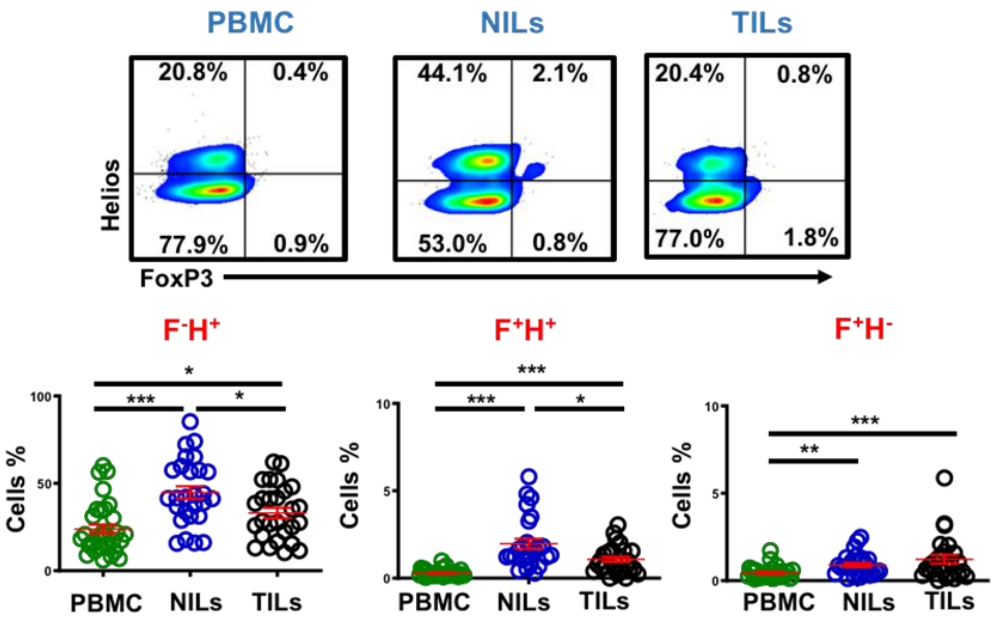

Figure S1. FoxP3 and Helios co-expression on CD8⁺ T cells in CRC patients. Representative flow cytometric plots and scatter plots show the levels of FoxP3⁻Helios⁺, FoxP3⁺Helios⁺ and FoxP3⁺Helios⁻, CD3⁺CD4⁻ (CD8+) T cells in PBMCs, NILs and TILs from CRC patients. Statistical significance levels indicated at * $p < 0.05$, ** $p < 0.01$ and *** $p < 0.001$.
